# Supplementary figures and images for: Genome-Wide Identification of TCP Gene Family in Dendrobium and Their Expression Patterns in Dendrobium chrysotoxum
Source: Int J Mol Sci. 2023 Sep 20;24(18):14320. doi: 10.3390/ijms241814320 (PMC10531990; doi:10.3390/ijms241814320)

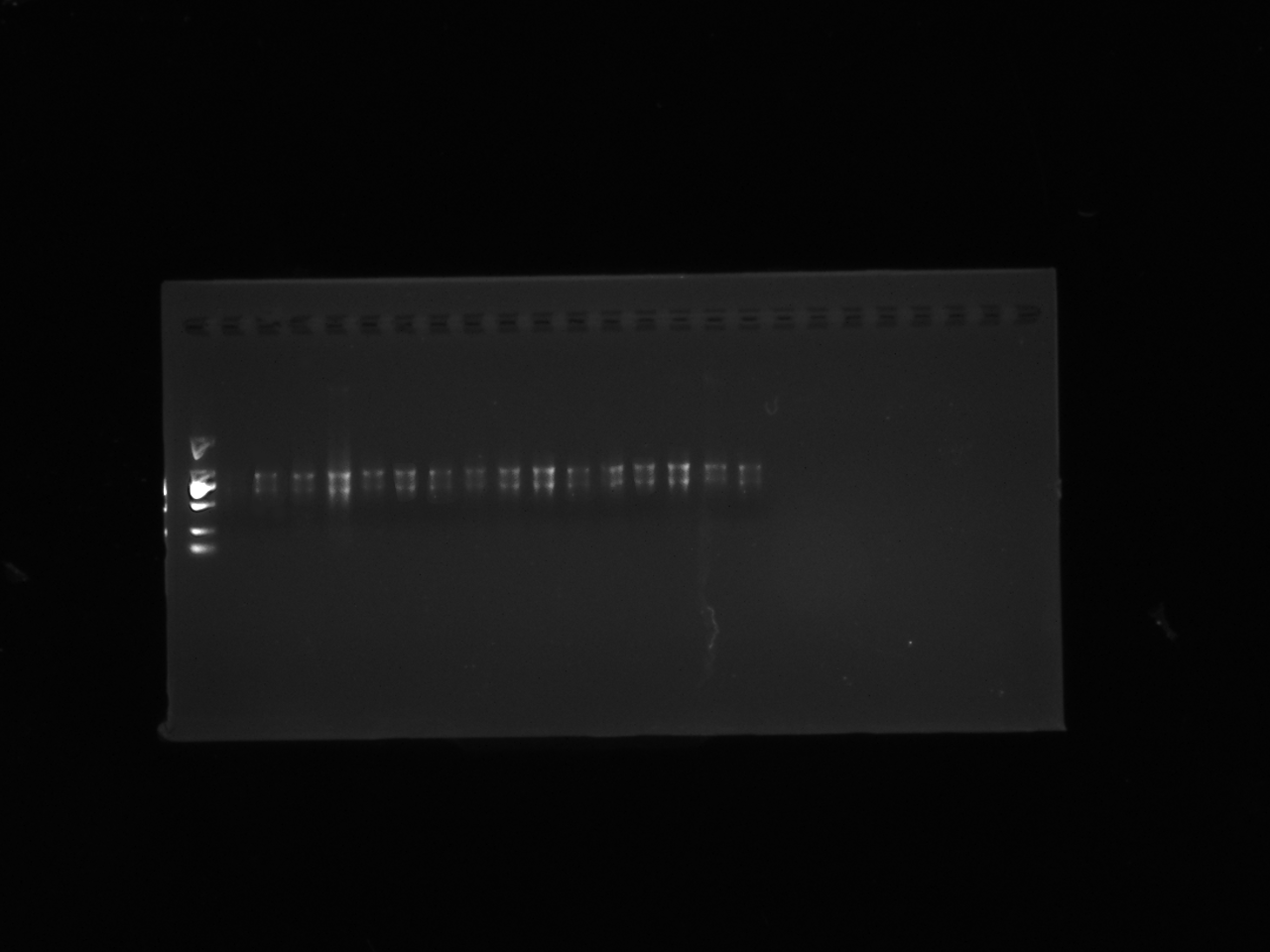

Supplement: Supplementary file 1 [file ijms-24-14320-s001.zip › Figure S1.png]

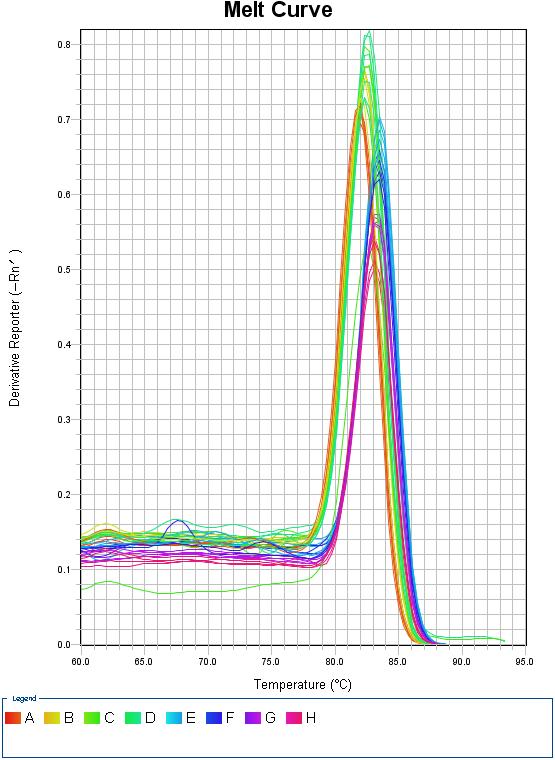

Supplement: Supplementary file 1 [file ijms-24-14320-s001.zip › qPCR Melt Curve/Melt Curve(1).jpg]

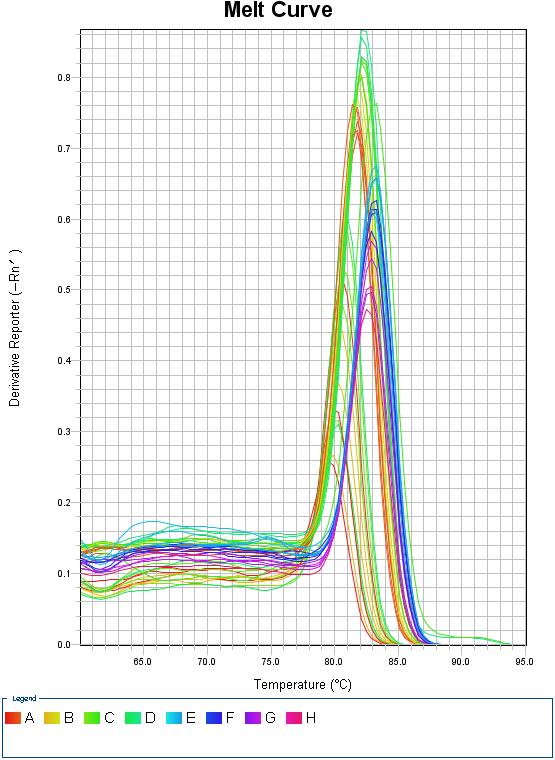

Supplement: Supplementary file 1 [file ijms-24-14320-s001.zip › qPCR Melt Curve/Melt Curve(2).jpg]

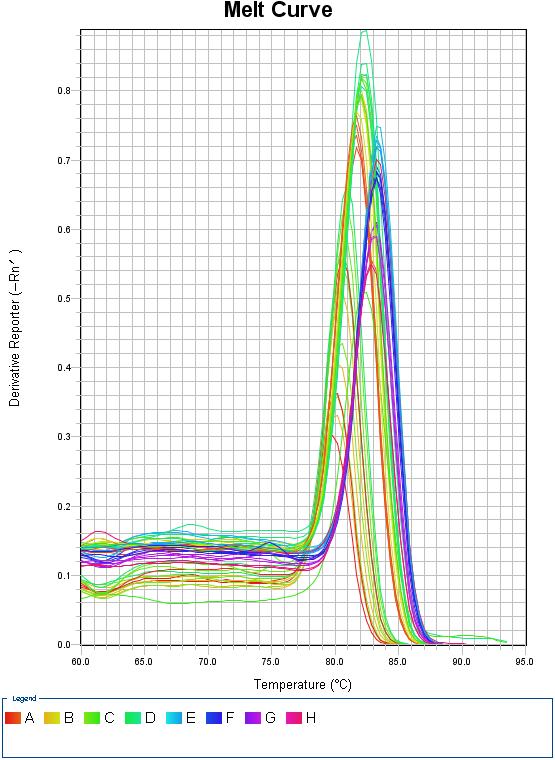

Supplement: Supplementary file 1 [file ijms-24-14320-s001.zip › qPCR Melt Curve/Melt Curve(3).jpg]

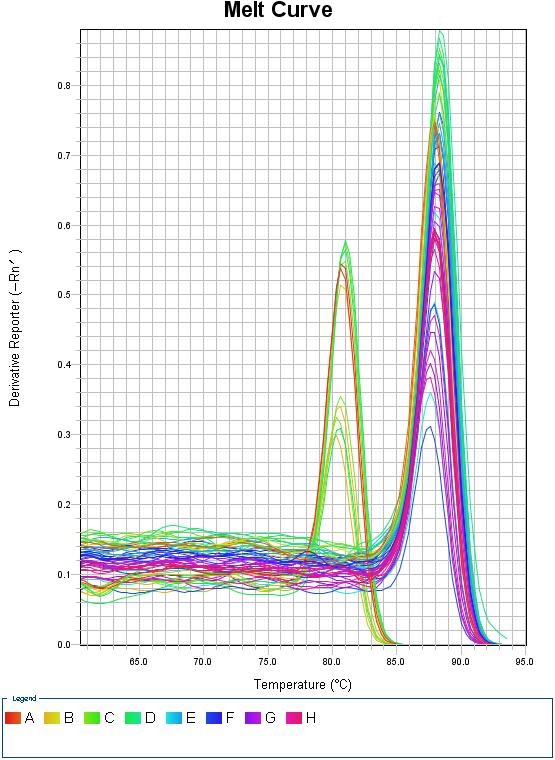

Supplement: Supplementary file 1 [file ijms-24-14320-s001.zip › qPCR Melt Curve/Melt Curve(4).jpg]
